# Design and Development of a Mobile App for Accessible Beach Tourism Information for People with Disabilities

**DOI:** 10.3390/ijerph16122131

**Published:** 2019-06-16

**Authors:** Diego Mayordomo-Martínez, Juan-Carlos Sánchez-Aarnoutse, Juan M. Carrillo-de-Gea, José A. García-Berná, José Luis Fernández-Alemán, Ginés García-Mateos

**Affiliations:** 1 Department of Structures, Construction and Graph Expression, Technical University of Cartagena, 30202 Cartagena, Spain; diego.mayordomo@upct.es; 2 Department of Information and Communications Technologies, Technical University of Cartagena, 30302 Cartagena, Spain; juanc.sanchez@upct.es; 3 Department of Computer Science and Systems, University of Murcia, 30100 Murcia, Spain; jmcdg1@um.es (J.M.C.-d.-G.); josealberto.garcia1@um.es (J.A.G.-B.); aleman@um.es (J.L.F.-A.)

**Keywords:** universal accessibility, physical disability, accessible tourism, mobile app, inclusive beaches

## Abstract

The global increase in the proportion of the population with disabilities has caused a greater awareness toward guaranteeing their use of public services. In particular, there is emphasis on the accessibility and inclusivity of tourism resources, to improve the enjoyment and well-being for people with motor disabilities. This paper presents a case study on accessibility to beaches in the Region of Murcia, Spain, which is one of the main tourist areas in the country. First, the most important elements that allow for the accessible use of beaches are analyzed and exposed in detail. Then, an extensive field-work in the area of interest has been carried out and its results are evaluated. Finally, the development of a new mobile app is described. The objective of this tool is to provide updated, accurate, and reliable accessibility information regarding the beaches. As a result, more than a third of the beaches analyzed had a high level of accessibility, while almost another third are totally inaccessible. The proposed application is a valuable tool, not only to help people with physical and motor disabilities, but also to raise awareness among local authorities to create and improve accessible services.

## 1. Introduction

According to the last update of the ad hoc module on the employment of people with disabilities by the “European Union labour force survey” [1], Europeans between the age of 15 and 64 years with disabilities represent ~45 million, i.e., 14% of the total population. Other international studies have estimated the world population with disabilities at 15% [2]. In Spain, where this research takes place, they constitute 8.5% of the inhabitants, which was reported by the Spanish National Institute of Statistics (INE) in November 2008 [3], and approximately 70% of them have some type of motor disability. This sector of the population usually needs assistance from support staff, thus raising the demand for accessibility information in their daily lives. In 2012, the INE reported that 69% of the people with motor disabilities face difficulties accessing leisure and cultural activities; 58% for leaving home, 41% for finding a job, 35% for entering public buildings, and 34% for using public transport [4]. Moreover, it is expected that by 2050 the population over 65 will increase in countries such as Spain by up to 34%, compared to 17% in 2000.

This group of people requires accessible and inclusive touristic resources free of barriers that prevent their use. Thus, it is essential that the information provided by public and private organizations is verified, clear, effective, and frequently updated. The present research is centered in the Region of Murcia, Spain, which is a touristic destination of the first level for both national and international tourists. This region stands out for its high-quality sun-and-beach tourism offer, which is its main claim. In this coastal province of the southeast of Spain, there are more than 200 beaches and coves, visited by more than 440,000 tourists every year [5].

In this context, it is clear that technology must play a central role in improving the well-being and integration of people with disabilities into society, providing tools to enhance accessibility to touristic and other types of services [6]. Over the last decade, the number of smartphones users has been increasing because of the advantages that these devices offer as compared to PCs and laptops, such as ubiquity, efficiency, portability, price, and rapidity in the execution of common tasks [7,8]. Therefore, mobile applications are, nowadays, an effective way to divulge any kind of information, especially when it is related to the location of places of public interest [9], as the case of accessible beaches for people with motor disabilities.

Currently, many research works can be found in the literature focused on the development of mobile applications for people with different types of disabilities or diseases. For example, McMahon et al. [10] presented an iPhone application for people with intellectual disabilities to teach them to identify food allergens using augmented reality. In a similar scenario, Ayres et al. [11] proposed a mobile app, also for the iPhone, to help students with mental disability and/or autism disorders. Srivastava et al. [7] developed a web responsive application of e-health tools for adolescents and young adults in India planned for use in mobiles. Other current applications of mobile technologies in the healthcare domain can be found in the review by Boulos et al. [12], which describes different uses in health, fitness, education, and ambient assisted living for people with chronic diseases.

Concerning the use of mobile applications in accessible tourism, Milicchio et al. [13] propose two apps for visiting archaeological sites both indoors and outdoors, in this case intended for people with auditory disability. Another application of accessible technology in a museum installation can be found in [14], for the Whitney Museum of American Art. More recently, Liu et al. [15] described an application for people in a wheelchair to find optimal travel plans. They implemented a program that uses linked data technologies in the area of touristic services and e-governance to create a smart city app for wheelchair accessibility. In [16], an application is presented for people with visual disability for the enjoyment of tourist services. Temes-Cordovez and Moya-Fuero [17] also developed a mobile app for barrier-free tourism in Valencia, in the southeast of Spain. The methodology used by the authors is based on systematic field-work, including accessibility information provided by the Municipality of Valencia. It is also worth mentioning the proposal of Potter et al. [18], which exposes the interest of virtual reality to nature-based tourism and its use by people with disabilities. Another example can be found in the app ACCEDE Murcia [9]. In this tool, detailed information about shop entrances for people with disabilities is depicted.

Generally speaking, in all these apps the main goal is to promote the elimination of barriers by providing information to people with disabilities. As observed in the literature, many apps are currently available; each one is related to a certain topic, exposing the importance of offering information in any field of interest for these people. Most of the works rely on data collected by technicians and specialized staff, although some of them allow users to update or incorporate new information. Finally, to the best of our knowledge, no previous work has focused on the accessibility of beach tourism.

Therefore, the objective of the present research is to analyze, design, and implement a new mobile application capable of providing updated and reliable information about the availability of accessible equipment and bathing assistance services, which are offered during the high season (i.e., in the summer months) in the area of interest. This research uses accessibility data of the Region of Murcia, provided by the Regional Plan of Accessibility to the Beaches, developed by the Tourism Department of the Region of Murcia and the Federation of regional associations of people with physical and organic disabilities of Murcia (FAMDIF).

The rest of the paper is organized as follows. Section 2 presents the accessibility equipment of the beaches, the definition of the field study and the app requirements. The obtained results, both from the point of view of the field study and the developed app, are described in Section 3, which is complemented with an evaluation and discussion of these results. Finally, the conclusions and future lines are drawn in Section 4.

## 2. Materials and Methods

This section describes the 3 main aspects of the present research. First, the existing equipment and services for accessibility to the beaches for people with motor disability are analyzed. Secondly, the planning and methodology used in the field-work are described. And finally, the requirements and tools applied in the development of the app are briefly introduced.

### 2.1. Definition of the Accessibility Elements to the Beaches

The inclusive accessibility of a tourist service—in our case a beach—is not a simple binary attribute that classifies it as “accessible” or “not accessible”. There are different dimensions and levels depending on the available equipment and the degree of accessibility in such tourist service. Hence, the first issue that arises is the analysis of the elements that allow the usage by people with motor or physical disability, that is, the equipment and services that inclusive beaches should provide and their classification according to a scale of usability and conservation.

For a complete compilation of these equipment and services, two sources of information were taken into account: national and regional legislation related to accessible tourism; and the opinion of the final users. Concerning the national legislation, the main source is given in the Article 9, “Urban beaches”, of Order VIV/561/2010 of the Spanish Ministry of Housing [19]. This order establishes that the municipalities are responsible for ensuring that all beaches totally or partially located in urban areas must have accessible points for all people, describing the required accessibility devices. On a regional level, the Regional Plan of Accessibility to the Beaches (PRAP-2006) developed in 2006 and updated in 2018, should be mentioned [20]. Regarding the opinion, judgment, and comments of the users with disabilities, this information has been obtained in collaboration with the regional organization FAMDIF. Through this collaboration, the final users indirectly participated in the definition, design, development, and validation of the app. FAMDIF is a federation composed of 17 associations, whose members add more than 3000 people. A standard process to collect periodically the opinion of the final users through surveys is not currently defined. The communication with them is carried out in two main ways: the feedback given at the request of the users through the corporate telephone and email of FAMDIF; and the periodical commissions of accessibility, where information and requests of all member associations are obtained. Therefore, the experience and knowledge of the managers and technical staff of FAMDIF indirectly collect all these contributions from the final users over the years.

**Figure 1:**
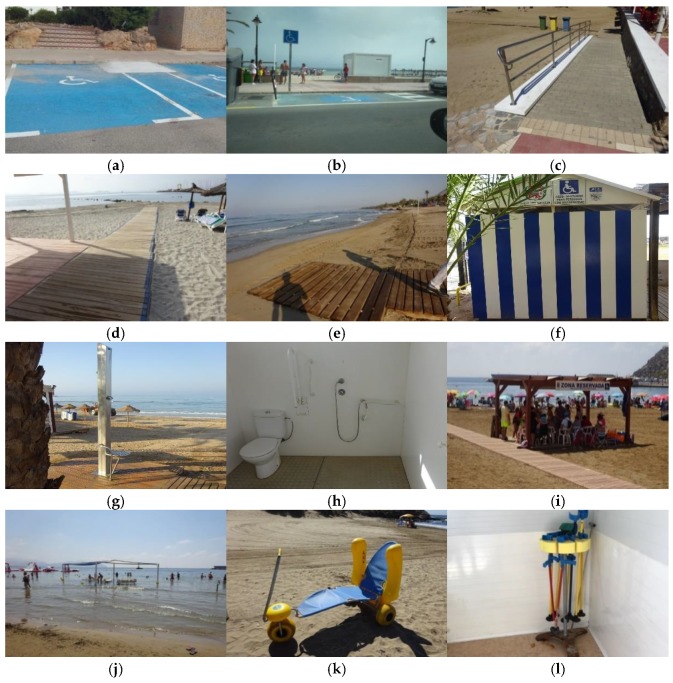
Examples of the different elements of accessibility to the beaches. (**a**,**b**) Parking reserved for people with disabilities. (**c**) Access ramp to the beach. (**d**) A walkway on the sand. (**e**) Roll-up gangway. (**f**) Adapted changing room. (**g**) Adapted shower. (**h**) Adapted toilet (and shower). (**i**) Adapted shaded area. (**j**) Marked bathing area. (**k**) Amphibious chair. (**l**) Amphibious crutches.

As a result of this compilation, the following relevant elements of accessibility to the beaches have been defined. Some examples of them are shown in Figure 1.

- Parking areas reserved for people with disabilities (Figure 1a,b). They must be 5 m long and 2.20 m wide, and have a contiguous transfer zone of 1.50 m wide for perpendicular parking, and 1.50 m in depth for parallel parking. They must allow access to the pedestrian pavement.
- Access ramps to the beach (Figure 1c). They are inclined planes, which exceed an inclination of 6%, or slopes of more than 20 cm. The ramps must have a width greater than 1.80 m and a length less than 10 m. The maximum longitudinal slope is 10% in sections up to 3 m, and 8% in sections up to 10 m.
- Walkways on the sand (Figure 1d). They are parts of the itinerary that pass through the sand and must be made with materials whose coefficient of thermic transmission allow people to walk barefoot.
- Roll-up gangways for the beach (Figure 1e). They are short paths appropriate to reach the border of the beach in a wheelchair.
- Adapted changing rooms (Figure 1f). They must be connected with an accessible itinerary, have a width in washbasins, showers, space for lockers, etc., greater than 1.20 m, and a turning space greater than 1.50 m, with doors wider than 0.80 m, sliding or open to the outside.
- Adapted showers (Figure 1g). They need a turning space of 1.50 m in diameter, with grab bars, mechanisms, accessories and support seats whose color is distinguished from the environment. The size of the area for wheelchair users is 0.80 × 1.20 m.
- Adapted toilets (Figure 1h). They must be connected with an accessible itinerary, have a turning space of more than 1.50 m, with doors wider than 0.80 m, sliding or open to the outside. As above, they must have grab bars and mechanisms, which contrast in color.
- Adapted shaded areas (Figure 1i). These areas allow the stay of the wheelchair users or their transference to an amphibious chair. The minimum area must be 2.50 m length and 1.80 m width, and the conditions have to be the same as the walkways.
- Bathing areas marked with buoys (Figure 1j). These are areas delimited in the water of the beach to the stay and bathing of people with disabilities.
- Amphibious chairs (Figure 1k). These chairs are technical support, which allow bath accessibility to people with disabilities, since they can move both on dry land and on the sea.
- Amphibious crutches (Figure 1l). Intended for people who need crutches to walk, this type of crutches can be conveniently used in the water.

Apart from the physical equipment, there are other interesting aspects to be considered, such as the services offered by the help staff:

- Type of help to the bathing. This service includes the kinds of assistance, which are given in the accessible points by the specialized staff.
- Calendar and timetable of help to the bathing. It is important that the services are provided in a broad and appropriate time range for people with disabilities. This element includes information about the time and dates when the service of help to the bathing is available.
- Adapted activities for people with disabilities. This item refers to all the additional activities that are available for entertainment on the beach.

All these items constitute the set of aspects that were inspected during the field-work performed by specialized technical staff. The list is complete, in the sense that it includes all the equipment that can be found in the current legislation, the additional elements indicated by the end users, and those found during previous field studies (although, evidently, it cannot be discarded that new ones will appear in the future). They can be classified considering the seaside stage experience in items to help: arriving to the beach; reaching the seashore; bathing in the sea; and stay on the beach. Table 1 contains a classification of accessibility elements in the four defined categories.

Finally, a general evaluation was also done for each beach, where they were classified according to the level of accessibility. Depending on the degree of compliance with the above requirements, these levels are defined as presented in Table 2, on a scale from 0 (lowest accessibility) to 9 (highest accessibility). Although these levels do not represent a continuous and homogeneous magnitude, they have been ordered in a scale of usability, according to the user experience: inaccessible beaches; accessible beaches with only one equipment (from the less to the more interesting); accessible beaches with two equipment; and beaches with all the equipment.

### 2.2. Design and Planning of the Field Study

After establishing the equipment and services that are desirable for inclusive beaches, an extensive field-work was planned and carried out by technical staff from FAMDIF. This field-work was done for the first time in 2004, and it has been updated annually since then, always before the beginning of the summer season.

**Figure 2:**
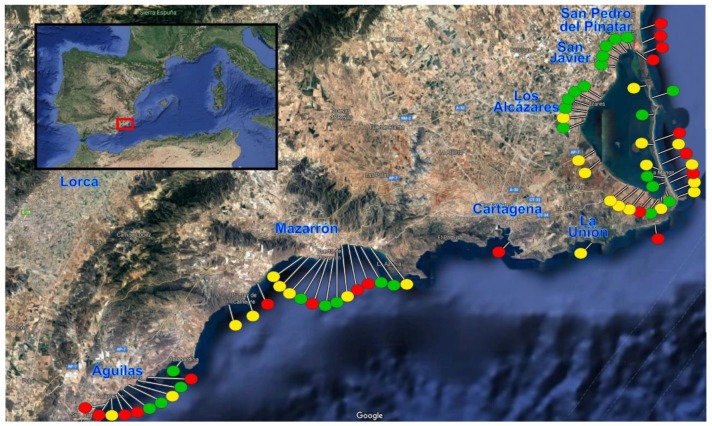
Geographical location of the beaches analyzed in the field study. The color of the circle indicates the resulting accessibility level: red (low, levels 0, 1, and 2); yellow (intermediate, levels 3 to 7); green (high, levels 8 and 9). Aerial views extracted from Google Maps.

During the 2018 update, a total of 68 beaches were visited and evaluated (four more than in the previous update); 54 of them are included in the regional plan PRAP-2006 [20]. The geographical location of these beaches is shown in Figure 2. They are separated into two main areas: beaches located on the southwest coast of the Region of Murcia; and beaches in Mar Menor and La Manga (the Mar Menor is a salt water lagoon of very high tourist density, separated from the Mediterranean Sea by a strip of land called La Manga).

**Figure 3:**
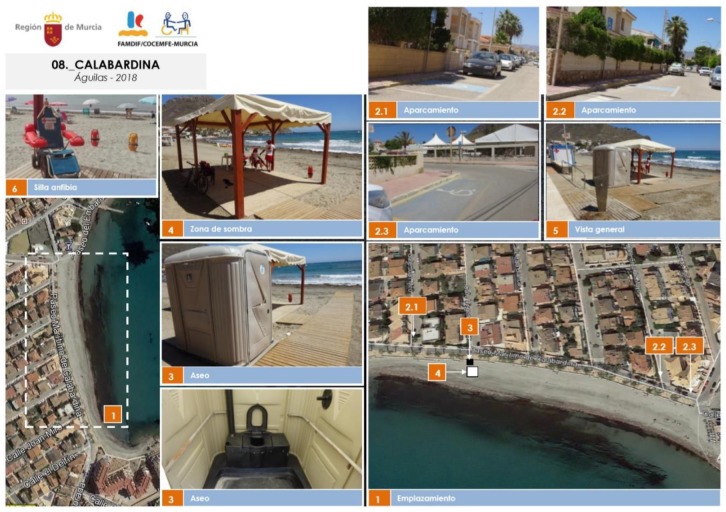
Sample graphical report obtained for the beach of Calabardina (in the municipality of Águilas) for the 2018 update of the field study. Image extracted from [21].

The procedure followed to update the field study for the 2018 summer season comprises the following phases:

- Visit and inspection of the beaches to assess accessibility services and equipment on-site. This process is done together with the technicians of the municipalities who are in charge of the accessibility to the beaches.
- General evaluation of the level of accessibility to all the beaches studied, on the scale from 0 to 9 defined in Table 2. A graphical report is also made for each beach, with pictures of the most significant accessibility elements. Figure 3 contains a sample image of these reports.
- Production of detailed tables of the beaches of each municipality. Then, a summary table is elaborated with the evaluation of the level of accessibility of all the beaches. The main incidents detected and possible improvements are included. This information is inserted in the specific tab of “Accessibility of existing beaches” in the website: www.murciaturistica.es.
- Elaboration of a summary table of the beaches according to the different levels of accessibility. This table distinguishes the two defined zones: beaches located on the southwest coast; and beaches in the areas of Mar Menor and La Manga.
- Geographical location of the beaches using aerial views of the coast. These pictures include images of the parking spaces reserved for people with disabilities and a general view of the accessible equipment available in the beach (shaded area, adapted bath and amphibious chair).

It is expected that this update process will be repeated in the future with an annual periodicity. In the following years, new beaches could be added to the field study. In any case, the beaches currently included in the app are more than 34% of the existing beaches, and all of them are the most important for the tourism sector of the Region of Murcia.

### 2.3. Design and Development of the Mobile Application 

The main technological development of this research is to create a new mobile application that allows the users to find accessible beaches in the Region of Murcia, offering all the related information obtained in the field study. The main requirements of this application were established together with FAMDIF members that provided, from their experience, the main barriers found when accessing to the beaches and the national and regional legislation consulted to know more about adequateness of the accesses to the beaches. These requirements are the following:

- The app should be designed for Android devices, since they have the highest share in the current market of portable devices, estimated around 75% in April 2019 [22]; moreover, in Spain, it is above 80%.
- The information shown in the app should be complete but avoiding overload. The elements reported for each beach are those described in subSection 2.1. The app should also contain pictures of the beaches.
- The app should be able to operate without the need to download data. In other words, it must work even if the device has no connectivity. This may be important in coastal areas that are not covered by a mobile network.
- Notwithstanding, the app should be able to update its information when the device gains connectivity. In this way, annual updates should be visible in the app without the need for user intervention.
- Finally, the app itself should be designed following the principles of usability and accessibility [23], being friendly, intuitive and easy to use.

Several innovative technologies have been used in the design and development of the app [24]. For software design and prototyping, the free tools Sketch (Bohemian B.V., The Hague, The Netherlands) and Invision (InVision, New York, NY, USA) were applied. They allow fast generation of views of the program, to obtain immediate feedback from the final users. Software development was done in Android Studio (Google, Mountain View, CA, USA) using Java, JavaScript and SQL programming languages. Several libraries were integrated into the development process: React-View-Model, OsmDroid (Open Street Map for Android), and SQLite database. Git (Software Freedom Conservancy) was used for the management and version control of the project, and XDE for software checking.

Three main use cases have been detected and implemented in the app: (1) access to the data through the list of municipalities; (2) quick search in the list of beaches; and (3) access to the list through the current geographical location. All these cases start with an action by the user, with the purpose of gaining information about a beach.

**Figure 4:**
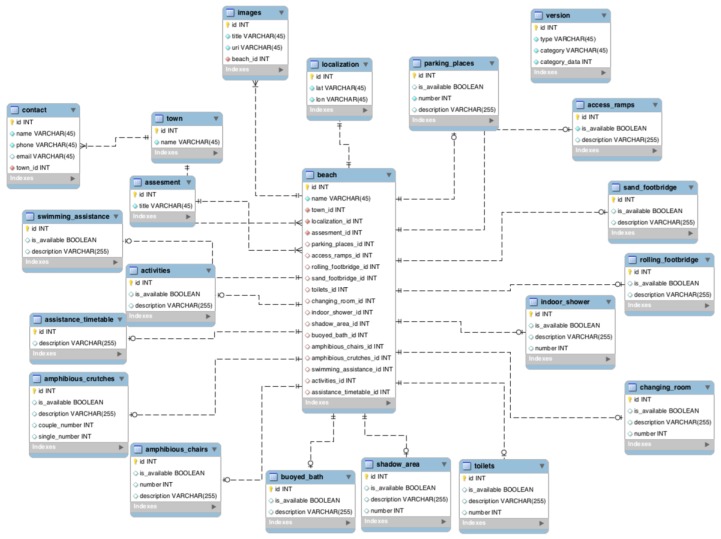
Entity-relationship diagram of the developed system [24].

Another important aspect of software development is the definition of the database. The information to be stored is described by an entity-relationship diagram, where each entity defines an object of interest with its name and attributes, and relationships are links between entities. The diagram defined in the present research is depicted in Figure 4. The core of the diagram is the beach entity, which is related to other entities representing different types of equipment, such as bathing assistance, amphibious chairs and shaded areas.

Since the time between information updates is long and there is a significant amount of data, it has been opted to distribute the data from 2018 with the app. In this way, when the app is downloaded, there will be information about the accessibility to the beaches available. This initial data is encapsulated in an SQLite database file. Updates to the database are checked through calls to the server when the app is launched, if an Internet connection is available. If the current version stored in the server is not found in the device, it is downloaded by the app. This method guarantees that the portable device only needs to download the information that has been modified.

## 3. Results and Discussion

The experimental results presented in this section consider the two main aspects of the work developed: the field study conducted, and the implemented app for accessibility information. Concerning the app, the execution of two sample use cases is briefly shown, and then a usability evaluation of the app is presented.

### 3.1. Results and Discussion of the Field Work

As indicated in Section 2, the field study was done on the 68 most important beaches of the Region of Murcia. A quick view of the results can be seen in Figure 2, where the classification of the beaches is shown in color (red, yellow, green). More detailed results are presented in Table 3.

In general, it can be deducted from Figure 2 that all municipalities are more or less concerned with accessibility, counting most of them with some inclusive beach for people with disabilities. The north-west coast of Mar Menor has a greater number of beaches with high accessibility, although none of them has adequate bathing assistance services (level 9). This fact contrasts with the data for 2017, where two beaches in this area provided these services [21,25]. There are 19 beaches inaccessible or poorly equipped (levels 0 to 2), representing 27.9% of the total beaches studied. To this percentage, the beaches and coves with less tourist relevance, which are mostly inaccessible, must be added.

On the other hand, 25 beaches were classified with high accessibility (levels 8 and 9), which is 36.9%. This number is very similar to the previous update, since the study is focused on beaches with the highest accessibility. In the intermediate levels, 24 beaches are partially equipped with some elements of accessibility (levels 3 to 7), being 35.2% of the total. In this category, the equipment found most often are the adapted toilet (in 21 of them) and the amphibious chair (in 15 beaches). However, only three of them have a shaded area, apart from the beaches in levels 8 and 9. Overall, there is no significant improvement in accessibility to the beaches in relation to the results of the field-work for the previous years [25].

**Figure 5:**
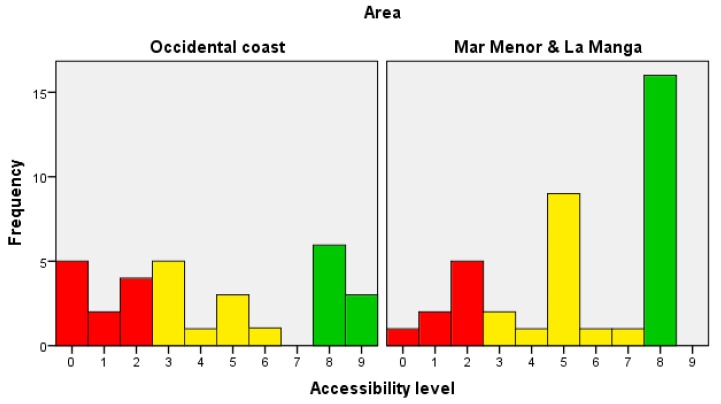
Histograms of accessibility divided by area to show differences between the beaches in the Occidental coast and Mar Menor & La Manga. Red: low; yellow: intermediate; and green: high.

A more detailed analysis was carried out to compare the accessibility level of the beaches located in the two areas under study: Occidental coast and Mar Menor & La Manga. Figure 5 shows the distribution of the data in the two samples using histograms.

An initial inspection of the graphs suggests that the beaches located in Mar Menor & La Manga have better accessibility level than those in the Occidental coast. A Mann–Whitney test (also known as the Wilcoxon test for independent samples) was carried out to find out whether the null hypothesis —i.e., equal population means—can be rejected. Let *diff* be:

*diff = mean(accessibility of Occidental coast) − mean(accessibility of Mar Menor & La Manga)* (1)

then, the null hypothesis is:

*H_0_: diff = 0* (2)

a one-tailed test was applied, so that the alternative hypothesis is as follows:

*H_A_: diff < 0* (3)

The results of the statistical test produced values of Mann-Whitney *U* = 445, *N*_1_ = 30, *N*_2_ = 38, and *p* = 0.058 one-tailed. This *p*-value indicates that the distribution in the two groups differs significantly (*p*-value < 0.1). With this statistical support, it can be said that the mean accessibility level of the beaches located in Mar Menor & La Manga is greater than that of the beaches located in the Occidental coast. Moreover, the median accessibility level in the Occidental coast is 3, while the median in Mar Menor & La Manga is 5. This situation could be explained due to the more touristic nature of the Mar Menor & La Manga area. For example, during the 2018 season, the Occidental coast had 72,642 visitors and 182,091 overnight stays, whereas Mar Menor & La Manga had 371,722 visitors and 1,472,817 overnight stays [5].

### 3.2. Sample Uses Cases of the Mobile Application

**Figure 6:**
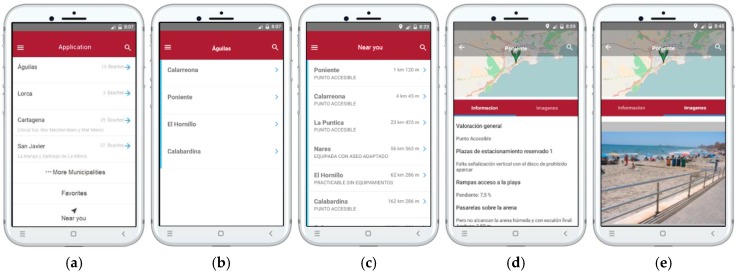
Sample screens of the proposed application [24]. (**a**) Initial screen. (**b**) List of beaches by municipality. (**c**) Search by proximity. (**d**) Information of a beach. (**e**) Images of a beach.

Two sample use cases of the app are presented in this subsection with the aim of graphically showing its use. Some views of the main screens of the app are depicted in Figure 6. As usual, the upper-left corner of the first screen (Figure 6a) contains the hamburger button that allows opening the main menu from any point. In the upper-right corner, the magnifying glass icon gives access to the quick search of beaches. The central area of the initial screen contains a list of the main municipalities, so in most cases, the user can access the desired information in a few clicks. The complete list is available in the button “More Municipalities”. Below are the options “Favorites” to show the beaches previously selected by the user, and “Near you” to search by distance.

#### 3.2.1. First Use Case: List of Municipalities

Consider that a person with a motor disability wants to spend their holidays in a certain city on the Murcian coast. Hence, the person needs to know the beaches where they could find adequate accessibility equipment. The search by municipality would be used in this case.

When the app is executed, the screen shown in Figure 6a appears. If the city is not in this list, the user would click on “…More Municipalities” to obtain the complete list. After selecting the corresponding city, the app will list all the accessible beaches of that municipality (Figure 6b). In this screen, beaches are given in geographical order, from west to east. By clicking on the name of the beach, the app will show all the relevant information (Figure 6d), including the map of the geographical location, the data collected in the field-work, and sample pictures of the equipment (Figure 6e). As expected, clicking the back icon returns to the list of beaches of the municipality.

In many cases, the information requested by the user can be obtained in just two clicks (select municipality and beach); and if the municipality is not in the initial list and the beach is in the bottom of the list, only four clicks would be needed. On the other hand, the information is presented in a simple and clear way, and all the components have the behavior that would be intuitively expected.

#### 3.2.2. Second Use Case: Search by Proximity

In this second use case, we suppose that the person with reduced mobility is on a trip along the coast of Murcia without a predefined destination, and the name of the municipality or nearby beaches are unknown. Therefore, the search by name is not useful, but the option of search by proximity would be used, by making a click on the “Near you” button of the initial screen. Next, the app gets the current GPS location of the device, and the nearest beaches are listed (Figure 6c). In this case, the list is sorted by the shortest distance; the current distance to each beach and a brief description of accessibility is shown in this screen. In this way, the person can get a quick idea of where to go.

Then, as in the previous use case, the user can select a name to access the screen of beach information and pictures (Figure 6d,e). Again, the entire process can be done in just 2 clicks. Finally, it can be observed that all the screens have the magnifying glass icon; this option would be used for a quick search when the name of the beach is already known.

### 3.3. Evaluation of the Usability of the Application

During the development of the application, aspects of usability were borne in mind to enrich the user’s experience. A total of two external experts in usability applied an evaluation method based on the usability inspection approach, or heuristic evaluation, with the purpose of evaluating the usability characteristics of the tool under study. Several user interface elements were analyzed, based on a set of usability principles for the development of applications proposed by Dix et al. [26]:

- **Predictability.** Support to determine the effect of future actions according to past interaction history.
- **Synthesizability.** Support to assess the effect of past operations on the current state.
- **Familiarity.** The extent to which the user’s knowledge and experience in other domains can be applied in a new system.
- **Generalizability.** Support to increase knowledge of specific interaction within and across applications to other similar situations.
- **Consistency.** Input–output behavior similarity from the same situations or equal task objectives.
- **Dialog initiative.** No dependency from artificial constraints on the input dialog in the system.
- **Multi-threading.** The capacity of the system to provide user interaction in more than one task at a time.
- **Task migratability.** The ability to perform a task to be internalized by the user or the system or shared between them.
- **Substitutivity.** The chance that the equivalent input and output values are substituted among themselves.
- **Customizability.** Possibility of user interface modification by the user or the system.
- **Observability.** Possibility to evaluate the internal state of the system concerning its perceptible representation.
- **Recoverability.** Ability to make corrective actions in case of making an error.
- **Responsiveness.** The fluidity perceived by the users of the system in operation.
- **Task conformance.** Degree in which services of the system comprise all the tasks that the users wish to carry out and in a way that is understandable.

These principles served as heuristics to perform the usability audit of the application. The evaluators punctuated each one of the principles based on the two use cases: list of municipalities and proximity search. After testing the functionalities offered by the system for each use case, a Likert scale was used to assess the usability principles of Dix, from 5 (totally agree) to 1 (totally disagree). Score 0 would be interpreted as the lack of facts to express an opinion. The results of the evaluation made by the experts are shown in Table 4.

It can be observed that the general level of usability is very high, with 66% of the criteria in levels 4 or 5. However, since the observers can sometimes agree or disagree simply by chance, the level of agreement between the two assessments was measured using Cohen’s kappa [27]. In this usability study, Cohen’s kappa weighted with Fleiss–Cohen coefficients (quadratics) was applied since these are ordered categories from two observers [28]. It was found that the interrater agreement value was kappa = 0.7762, 95% CI (0.5099, 1), which represents a substantial level of agreement [29]. Evaluation of the final users is still not available, since the app is in a phase of distribution and evaluation.

## 4. Conclusions

Information and communication technologies are called upon to play a fundamental role in the well-being and integration into society of people with disabilities and chronic diseases. To that end, new tools and applications should be made available to help them in their daily lives. In this article, a complete study of the accessibility to beaches in the Region of Murcia is presented, complemented with the design and development of an innovative application, for people with mobility disabilities, that contains updated, detailed, and reliable information, which has been obtained by architects and technical architects of the regional organization FAMDIF.

In general, a high level of accessibility has been observed, with 36.9% of the beaches in the top levels (levels 8 and 9). Only 27.9% cannot be accessed or enjoyed by people in a wheelchair (levels 0 to 2). However, there are many beaches at intermediate levels, 35.2%, partial but not fully equipped. Significant statistical differences were found between the two defined study areas, being the most relevant touristic areas the more inclusive. These large differences, among other factors related to visibility and popularity, can eventually have an impact on funding availability to carry out service improvement activities. In turn, these differences might, to some extent, account for the differences in the accessibility of the beaches found. In this sense, we think that the availability of information with the proposed app will serve to increase the awareness of local authorities to improve the accessibility of the services.

In relation to the use of the app, it can be seen that the program is complete, intuitive, easy to use, and has been developed in compliance with the guidelines of usability and good practices. The usability analysis shows a high compliance of the criteria, with all the scores above 3, except for the possibility of modifying the interface by the user since the ease of use must prevail in an app like this. In the future, we hope that by continuous information updates, the inclusion of new beaches (until approaching all of them), and the feedback from the final users, the system will be able to improve and promote the well-being of people with disabilities. Involving citizens in the use of the application could also have an impact on the demand for more information about accessibility to the beaches. In this context, a commitment from the authorities could speed up the database growth in order to provide potential users with more information they require from the city councils prior to organizing their leisure time.

Another limitation of the present research that will be addressed in the future is the accessibility of the application itself by people with different types of disabilities. In many cases, people with disabilities suffer from multiple difficulties, such as visual or intellectual disorders, so the development of the system should also consider them in order to be fully inclusive.

## Figures and Tables

**Table 1:** Classification of the elements of accessibility to the beaches.

Accessibility Element	Arriving to the Beach	Reaching the Seashore	Bathing in the Sea	Stay on the Beach
Physical equipment				
Parking areas reserved	×			
Beach access ramps		×		
Walkways on the sand		×		
Roll-up gangways		×		
Adapted changing rooms				×
Adapted showers				×
Adapted toilets				×
Adapted shaded areas				×
Bathing areas with buoys			×	
Amphibious chairs			×	
Amphibious crutches			×	
Services				
Help to the bathing			×	
Help calendar				×
Adapted activities				×

**Table 2:** Definition of the levels of accessibility to the beaches.

Accessibility Level	Description
0. Not accessible	Beach that cannot be used by people with motor disabilities with autonomy and security.
1. Usable but without equipment	Beach that only has a ramp and a walkway on the sand, but no additional equipment.
2. Equipped but not usable	Beach that has some adapted equipment, but there are steps or short walkways, which impede their use by people with disabilities.
3. Equipped with adapted toilet	Beach with adapted toilet, but without an amphibious chair or shaded areas.
4. Equipped with amphibious chair	Beach with an amphibious chair, but not an adapted toilet or a shaded area.
5. Equipped with adapted toilet and amphibious chair	Beach with adapted toilet and amphibious chair, but without a shaded area.
6. Equipped with adapted toilet and shaded area	Beach with an adapted toilet and a shaded area to stay, but without an amphibious chair.
7. Equipped with shaded area and amphibious chair	Beach with shaded area and amphibious chair, but without an adapted toilet.
8. Accessible point	Beach with reserved parking, adapted toilet, walkways, amphibious chair, and shaded area, but without services to help the bathing.
9. Accessible point with assisted bathing	Beach with all the equipment indicated above, and also services to help the bathing with amphibious chair given by specialized staff.

**Table 3:** Number of beaches in each accessibility level and in each geographical area, obtained from the field study for the 2018 update. The levels of accessibility are defined in Table 2.

Accessibility Level	0	1	2	3	4	5	6	7	8	9
**Occidental coast (N_1_ = 30)**	5	2	4	5	1	3	1	0	6	3
**Mar Menor & La Manga (N_2_ = 38)**	1	2	5	2	1	9	1	1	16	0
**Total beaches (N = 68)** **(%)**	6 8.8%	4 5.9%	9 13.2%	7 10.3%	2 2.9%	12 17.6%	2 2.9%	1 1.5%	22 32.4%	3 4.5%

**Table 4:** Evaluation of the principles of Dix used in the usability audit of the developed app.

Usability Principles of Dix	Use Case 1: List of Municipalities		Use Case 2: Proximity Search	
	Evaluator A	Evaluator B	Evaluator A	Evaluator B
**Predictability**	4	5	5	5
**Synthesizability**	5	4	4	4
**Familiarity**	5	5	5	4
**Generalizability**	4	4	5	4
**Consistency**	3	4	4	4
**Dialog initiative**	3	3	4	3
**Multi-threading**	3	3	3	3
**Task migratability**	4	3	5	4
**Substitutivity**	3	3	2	2
**Customizability**	2	2	2	2
**Observability**	3	3	3	3
**Recoverability**	3	4	4	4
**Responsiveness**	4	5	5	5
**Task conformance**	5	4	4	4
**Predictability**	4	5	5	5
**Synthesizability**	5	4	4	4
